# Two-Photon-Excited FLIM of NAD(P)H and FAD—Metabolic Activity of Fibroblasts for the Diagnostics of Osteoimplant Survival

**DOI:** 10.3390/ijms25042257

**Published:** 2024-02-13

**Authors:** Tatiana B. Lepekhina, Viktor V. Nikolaev, Maxim E. Darvin, Hala Zuhayri, Mikhail S. Snegerev, Aleksandr S. Lozhkomoev, Elena I. Senkina, Andrey P. Kokhanenko, Kirill A. Lozovoy, Yury V. Kistenev

**Affiliations:** 1 Laboratory of Laser Molecular Imaging and Machine Learning, Tomsk State University, Lenin Ave. 36, 634050 Tomsk, Russia; tatiana.lepekhina@mail.tsu.ru (T.B.L.); vik-nikol@bk.ru (V.V.N.); hala.zuhayri@mail.tsu.ru (H.Z.); snegerev@mail.tsu.ru (M.S.S.); elena.senkina.1995@mail.ru (E.I.S.); yuk@iao.ru (Y.V.K.); 2 Independent Researcher, 10178 Berlin, Germany; maxim.darvin@protonmail.com; 3 Institute of Strength Physics and Materials Science of the Siberian Branch of the Russian Academy of Sciences (ISPMS SB RAS), 634021 Tomsk, Russia; asl@ispms.tsc.ru; 4 Department of Quantum Electronics and Photonics, Faculty of Radiophysics, National Research Tomsk State University, Lenin Av. 36, 634050 Tomsk, Russia; kokh@mail.tsu.ru

**Keywords:** two-photon microscopy, fluorescence lifetime imaging, phasor plot, metabolic activity, glycolysis, oxidative phosphorylation, NAD(P)H, FAD, porous ceramics, fibroblasts

## Abstract

Bioinert materials such as the zirconium dioxide and aluminum oxide are widely used in surgery and dentistry due to the absence of cytotoxicity of the materials in relation to the surrounding cells of the body. However, little attention has been paid to the study of metabolic processes occurring at the implant–cell interface. The metabolic activity of mouse 3T3 fibroblasts incubated on yttrium-stabilized zirconium ceramics cured with aluminum oxide (ATZ) and stabilized zirconium ceramics (Y-TZP) was analyzed based on the ratio of the free/bound forms of cofactors NAD(P)H and FAD obtained using two-photon microscopy. The results show that fibroblasts incubated on ceramics demonstrate a shift towards the free form of NAD(P)H, which is observed during the glycolysis process, which, according to our assumptions, is related to the porosity of the surface of ceramic structures. Consequently, despite the high viability and good proliferation of fibroblasts assessed using an MTT test and a scanning electron microscope, the cells are in a state of hypoxia during incubation on ceramic structures. The FLIM results obtained in this work can be used as additional information for scientists who are interested in manufacturing osteoimplants.

## 1. Introduction

Biomedical surgical osteoimplants are commonly used in tissue engineering [1]. The success of implantation is related to the implant survival and healthy state of the surrounding tissue [2,3]. The survival rate of implants depends on factors related to the surgical and clinical features of the installation of osteoimplants, but one of the main ones is the composition and properties of the material from which they are made [4]. Non-toxicity and biocompatibility are important characteristics of the materials used for the manufacture of osteoimplants, but, recently, special attention has been paid to the development of materials with bioresorption [5,6]. This is because, over time, the damaged area of bone tissue is capable of self-healing, and only temporal support is needed to preserve the integral structure of the bone. However, bioresorption is poorly controlled, and, most often, the decomposition of the implant occurs faster than the restoration of bone tissue [7]. Bioinert materials can result in a more reliable integral design in the implant–bone area. They are not capable of decomposition, but they do not have toxicity or chemical activity in the environment of a living organism, and they have the necessary physical and mechanical characteristics. The most important bioinert materials used for biomedical purposes are ceramics based on aluminum oxide (Al_2_O_3_) and zirconium dioxide (ZrO_2_), which are stable, non-toxic, and biologically inert [8]. Because of their good corrosion resistance and biocompatibility properties, Al_2_O_3_ and ZrO_2_ ceramics are the most important ceramic oxides that are used for the reconstruction and replacement of damaged bone and joint tissues, in total hip and knee arthroplasty [9]. Studies indicate that chromium-alloyed zirconia material hardened with Al_2_O_3_ is non-toxic and do not show any long-term pathogenic effect in vivo, which justifies the use of this material as dental and orthopedic implants [10]. ZrO_2_ is widely used as a material in femoral heads for orthopedic hip implants [11].

For biocompatibility and viability analyses, cell colonies can be cultured on a specific test material, where cell proliferation and adhesion can be assessed [3]. Depending on the substrate surface profile, the cellular activity can either increase or decrease [12]. It has been noted that metabolic activity and cell adhesion can decrease due to an increase in the surface roughness of the bacterial poly (3-hydroxybutyrate-co-3-hydroxyvalerate) copolymer on which NIH/3T3 mouse fibroblasts were incubated [13]. It has been shown that the surface roughness (porosity) of the implants can improve the process of osteogenation compared to relatively smooth surfaces [14]. This is because the pores provide a stable matrix for cell attachment and osteogenic factors. The strength of the implant attachment increases due to the development of bone tissue inside the pores.

The interaction of the cells with the surrounding exogenous substances/materials (the implant surface) may lead to the development of inflammation, which is accompanied by the generation of free radicals that affect the redox and antioxidant status of the cells [15,16]. Therefore, the determination of the cell’s redox state can serve as a marker for the development of inflammation and implant survival. Modern microscopy techniques allow us to analyze the cell metabolic activity, as well as the biocompatibility of the cell lines and the implant biomaterials. The fluorescence intensity and lifetime decay of flavin adenine dinucleotide (FAD), nicotinamide adenine dinucleotide (NAD), and nicotinamide adenine dinucleotide phosphate (NADP) provide information about the cell’s metabolic activity. The fluorescent properties of the reduced form of NAD (NADH) and its phosphorylated form, NADPH, overlap; thus, the source of their combined fluorescence is denoted NAD(P)H [17]. NAD(P)H and FAD are involved in various redox reactions in the cell, ATP formation, and antioxidant reactions [18]. NAD is involved in the major pathways of energy production such as fatty acid oxidation and glycolysis [19]. In cells, FAD acts as an enzyme cofactor in redox processes [20]. FAD participates in lipid peroxidation, antioxidant reactions, and oxidative phosphorylation [18].

NAD(P)H and FAD are present in cells in two states, free and bound [21]. The bound NAD(P)H form associated with various proteins is localized in the intermembrane space of the mitochondria and is responsible for the processes of oxidative phosphorylation [22,23]. At the same time, the fluorescence lifetime of protein-bound NAD(P)H depends on the enzyme with which it is bound [24,25]. It was noted that the protein-bound form of NAD(P)H emits with a higher intensity and has a higher quantum yield than the free form [26]. The NAD(P)H free form is located in the cell cytosol and is involved in glycolysis process [27].

Fluorescence microscopy can be used to assess cellular metabolism by imaging endogenous fluorophores [20,28,29]. The metabolic status can be determined by the integral characteristic of fluorescence excited at different wavelengths and by estimating the fluorescence lifetime decay [30]. The fluorescence lifetime imaging microscopy (FLIM) approach allows the imaging of NAD(P)H and FAD with a high spatial resolution, determining their free and bound states without the need of staining [31]. NAD(P)H is fluorescent and has an excitation maximum at ≈340 nm and an emission maximum at ≈450 nm [32]. However, when NAD(P)H binds to proteins, the fluorescence quantum yield changes [33]. The excitation maxima of the oxidized form of FAD are in the range of wavelengths between 360 and 450 nm; the emission maximum is at ≈520 nm [34].

The bound FAD and free NAD(P)H forms correspond to a shorter fluorescence lifetime, while a longer fluorescence lifetime corresponds to free FAD and bound NAD(P)H. The fluorescence lifetime in the cells is determined by NAD(P)H (0.3–2.0 ns) and FAD (0.3–2.7 ns) [27]. FAD is synthesized as a result of oxidative phosphorylation, while the NAD(P)H cofactor is synthesized during glycolysis; a decreased FAD/NAD(P)H redox ratio indicates a shift in the balance of energy metabolism from oxidative phosphorylation towards glycolysis and intensive cell metabolism. A shift in cell metabolism towards oxidative phosphorylation occurs when the FAD/NAD(P)H redox ratio is increased [35].

The aim of the present work is to develop a diagnostic method for the assessment of the toxicity of ceramic structures as materials for osteoimplants induced on the fibroblasts near the implant surface based on an analysis of the shift in cells’ metabolic activity by using two-photon microscopy and FLIM.

## 2. Results

### 2.1. Fluorescence Microscopy of NAD(P)H and FAD

The fluorescence lifetime decay components $a_{1}$, $a_{2}$, $\tau_{1}$, $\tau_{2}$, and $\tau_{m}$ were determined for NAD(P)H using two-photon excitation at 760 nm, as presented in Table 1.

**Figure 1:**
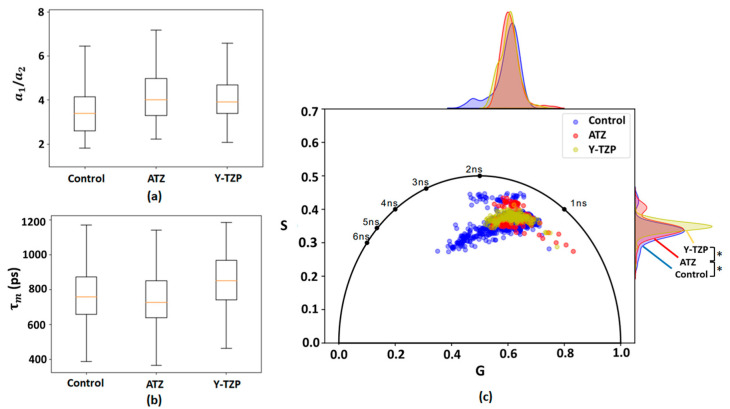
The ratio of the $a_{1}$ and $a_{2}$ amplitudes (**a**) and the mean fluorescence lifetime $\tau_{m}$ (**b**) of the 3T3 fibroblasts (two-photon excitation at 760 nm; fluorophore is NAD(P)H) incubated on ATZ, Y-TZP, and control glass; and (**c**) phasor plot of the 3T3 fibroblasts incubated on ATZ (red), Y-TZP (yellow), and control glass (blue) (two-photon excitation at 760 nm, fluorophore is NAD(P)H);*—significance level of *p* < 0.05.

It can be concluded that the $\tau_{1}$, $\tau_{2}$, and $\tau_{m}$ for Y-TZP are higher compared to the control and ATZ groups. The amplitude of the short component $a_{1}$ has a greater contribution compared to the long component $a_{2}$. This fact is confirmed by the ratio $a_{1}$/$a_{2}$, which is also presented in Table 1. The ratio of amplitudes ($a_{1}$/$a_{2}$) and $\tau_{m}$ are presented in Figure 1a,b.

In order to understand the changes that occur during the incubation of 3T3 fibroblasts on ATZ and Y-TZP ceramic surfaces, a phasor plot was constructed taking into consideration the fluorescence lifetime and amplitudes (short and long) of cells, as presented in Figure 1c. On the phasor plot, the fluorescence lifetimes in all groups overlap with each other and are located approximately in the same phasor area. However, in the control group of 3T3 fibroblasts, a shift towards a longer fluorescence lifetime was observed, whereas, in 3T3 fibroblasts incubated on ATZ and Y-TZP, a shift towards a shorter fluorescence lifetime was observed. The control, ATZ, and Y-TZP samples were pairwise distinguishable on the S variable with a significance level of *p* < 0.05 (see Figure 1c).

The fluorescence lifetime decay components $a_{1}$, $a_{2}$,$\;a_{1}$/$a_{2}$, $\tau_{1}$, $\tau_{2}$, and $\tau_{m}$ were determined for two-photon excitation at 830 nm (FAD), as presented in Table 2.

**Figure 2:**
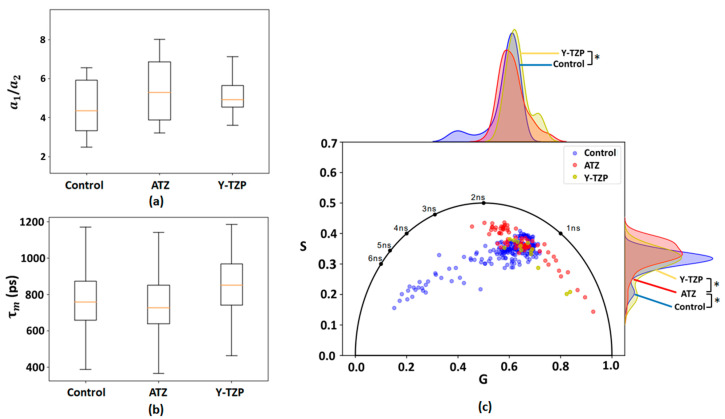
The ratio of the $a_{1}$ and $a_{2}$ amplitudes (**a**) and the mean fluorescence lifetime $\tau_{m}$ (**b**) of the 3T3 fibroblasts (two-photon excitation at 830 nm; fluorophore is FAD) incubated on ATZ, Y-TZP, and control glass; and (**c**) phasor plot of the 3T3 fibroblasts incubated on ATZ (red), Y-TZP (yellow), and control glass (blue) (two-photon excitation at 830 nm; fluorophore is FAD); *—significance level of *p* < 0.05.

From Table 2, it can be concluded that $\tau_{1}$, $\tau_{2}$, and $\tau_{m}$ for ATZ are higher compared to the control and Y-TZP groups. The amplitude of the short component $a_{1}$ has a greater contribution compared the long component $a_{2}$. There is a higher $a_{1}$/$a_{2}$ ratio in the control group compared to ATZ and Y-TZP; no statistical significance was found between all groups. The ratio of amplitudes ($a_{1}$/$a_{2}$) and $\tau_{m}$ were obtained as presented in Figure 2a,b.

The distribution of the free/bound FAD ratio was determined in 3T3 fibroblasts incubated on ATZ and Y-TZP ceramics and control glass and is presented on the phasor plot in Figure 2c. An analysis of free and bound FAD on the phasor plot shows that, compared to control, 3T3 fibroblasts incubated on Y-TZP showed a significant shift towards a higher value of the G component, but, for ATZ, the changes are not significant. The normality tests for the FAD samples revealed that the Y-TZP sample had a normal distribution for the G variable. Moreover, the control and Y-TZP samples were pairwise distinguishable on the G variable with a significance level of *p* < 0.05 (see Figure 2c). Pairwise differences in the S variable were observed between the control and ATZ samples, as well as between the ATZ and Y-TZP samples.

**Figure 3:**
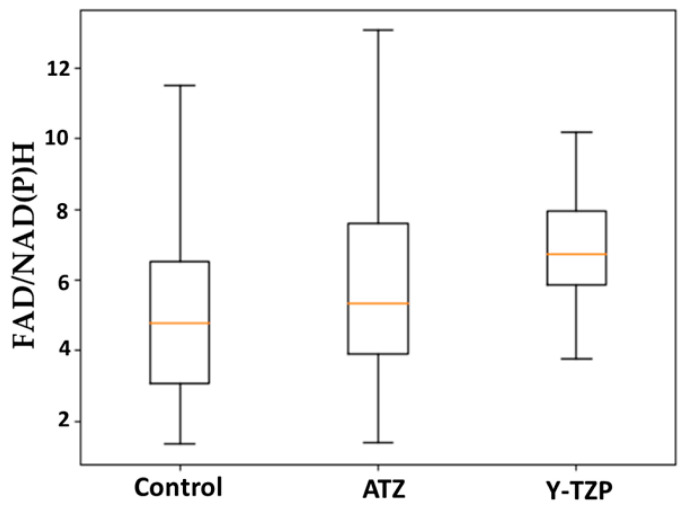
Redox status of 3T3 fibroblasts incubated on Y-TZP, ATZ, and control glass determined by the FAD/NAD(P)H ratio. No statistical significance was found between all groups.

The redox status of 3T3 fibroblasts incubated on Y-TZP, ATZ, and control glass determined by the FAD/NAD(P)H ratio is shown in Figure 3.

The control group has the lowest FAD/NAD(P)H redox ratio between the compared groups. However, fibroblasts incubated on Y-TZP and ATZ exhibited a trend (*p* > 0.05) towards a higher redox ratio compared to the control group. The highest redox ratio was observed in the Y-TZP group.

### 2.2. Determining Fibroblast Viability Using the MTT Test

**Figure 4:**
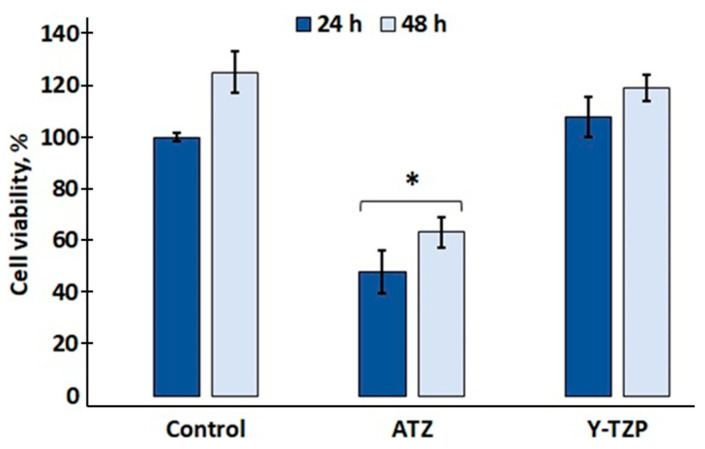
Cell viability (MTT test) after 24 (dark blue) and 48 h (light blue) of incubation on ATZ and Y-TZP ceramic and control glass surfaces. * *p* < 0.05 compared with the control group.

The results of fibroblast viability (MTT test) are presented in Figure 4. The number of cells cultured for 24 h on the glass coverslip was 100%. When incubated for 24 h on ATZ ceramic, fibroblast viability was 50%, and 108% on Y-TZP ceramic. Further incubation (48 h in total) shows an increase in the number of fibroblasts in the control group and in both the ATZ and Y-TZP groups. This indicates that the incubated 3T3 fibroblasts actively proliferate on the control glass and on the ATZ and Y-TZP ceramic surfaces. The MTT test was used primarily for comparison with FLIM results.

### 2.3. Light Microscopy and SEM Images of the Surface Ceramic Samples

**Figure 5:**
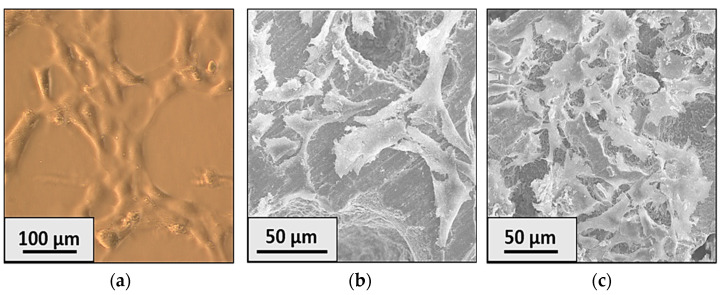
Image obtained by light microscopy for 3T3 fibroblasts incubated on a control glass (**a**); and SEM images of fibroblasts incubated on ATZ (**b**) and Y-TZP (**c**) surfaces.

In addition to the MTT test, fibroblast morphology can be checked using light microscopy and SEM images. The 3T3 fibroblasts on the plate glass coverslip processed to enhance cell adhesion are shown in Figure 5a. The fixation of the fibroblasts was carried out on day 5. Knowing the morphological features of the fibroblasts, we can give an initial assessment of the toxicity induced by the material. Analyzing the light microscopy and SEM images, we can conclude that the fibroblasts are alive and proliferate well on all structures. Under the appropriate conditions, 3T3 fibroblasts have a flattened, spindle-like shape with elongated lamellipodia—typical for fibroblast morphology in the tissue [36]. A similar morphology is observed on zirconia ceramics, which, once again, confirms the non-toxicity of the ATZ and Y-TZP materials.

## 3. Materials and Methods

### 3.1. Ceramic Preparation

**Figure 6:**
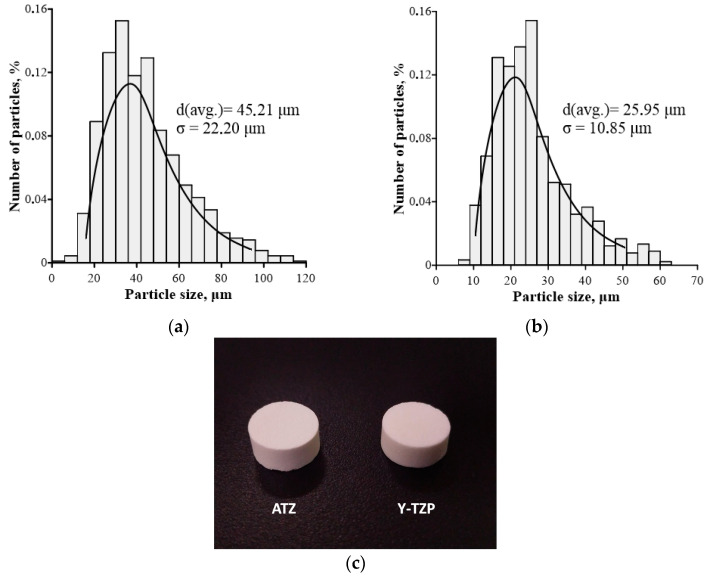
Size distribution of (**a**) Y-TZP and (**b**) ATZ particles; and photo of ATZ (**c**, left) and Y-TZP (**c**, right) ceramic samples.

The initial compositions of ceramics were prepared by mechanical mixing of powders: 80% ZrO_2_ + 3 mol.% Y_2_O_3_ + 20% Al_2_O_3_ (yttrium-stabilized zirconia ceramics hardened with aluminum oxide—ATZ) and ZrO_2_ + 3 mol.% Y_2_O_3_ (stabilized zirconia ceramics—Y-TZP) (Tosoh, Tokyo, Japan), UHMWPE particles from Jiangxi (China) with a size of 150 µm, and rosin—Pinus Brazil (Brazil) [37,38]. Spherical ultra-high-molecular-weight polyethylene (UHMWPE) particles with an average size of 150 μm were chosen as pore-forming particles. The particle content was 50 vol.% of the total powder mixture volume. The compositions used in the study are included in the ISO 13356-2016 and ISO 6474-2:2012 registers as materials with high biological inertness. The biocompatibility of the ceramics used has been demonstrated by various scientific groups [39,40]. The samples were compacted by cold uniaxial pressing in a steel mold under a pressure of 130 MPa (IP-500-1, Tochmashpribor, Armavir, Russia). Pore-forming particles were removed by annealing in an air furnace (LHT 08/18/3310, Nabertherm, Lilienthal, Germany) and sintering at a temperature of 1600 °C, at a heating rate of 160 °C/h and with a holding time of 1 h. The obtained samples have a cylindrical shape with a height of 5 mm and a diameter of 10 mm. The average particle size of ATZ and Y-TZP powders was 25.95 μm and 45.21 μm, respectively. (Figure 6a,b). Rosin was sifted step by step through sieves with different mesh sizes. Particles with sizes ranging from 350 to 500 µm were selected for the experiment. The shrinkage of the samples after sintering was about 50%. The test procedures for ceramic samples and cells are presented in Table 3. Example photo of ATZ and Y-TZP ceramic samples is shown in Figure 6c.

### 3.2. Cell Culture

The study was conducted using mouse embryonic fibroblasts of the 3T3 cell line, which is widely used in biomedical research [41]. According to ISO 10993-5—2011 standards (https://www.russiangost.com/p-61582-gost-iso-10993-5-2011.aspx, access on 20 December 2023), the toxicity of the material is studied by direct contact using known cell cultures that can be easily transplanted in laboratory conditions. Cells that differentiate into osteoblasts, bone cells, are not capable of long-term cultivation, since their calcification mechanism is triggered over time. The 3T3 mouse fibroblasts used in the study are part of the ISO Recommended Cell Culture Registry. Possessing high sensitivity, fibroblasts allow an accurate assessment of the toxicity of a material in the primary analysis. Cells were cultured in a 24-well plate (Corning, Glendale, CA, USA) in DMEM/F-12 nutrient medium (BioloT, Saint Petersburg, Russia) with the addition of 10 wt.% fetal bovine serum (FBS) (BioloT, Saint Petersburg, Russia) and 5 wt.% antibiotic (streptomycin) (BioloT, Saint Petersburg, Russia). A Sanyo MCO-5AC incubator (Sanyo, Osaka, Japan) at a temperature of 37 °C and air atmosphere at 5% CO_2_ was used. Subsequently, cells were passaged after trypsinization using a 1:1 Trypsin-Versene solution (BioloT, Saint Petersburg, Russia) and Dulbecco’s phosphate-buffered saline (DPBS) (BioloT, Saint Petersburg, Russia). Cells were planted on the outer surface of the scaffolds and incubated in a nutrient medium. The concentration of cells used for cultivation was 50,000 cells/mL and was the same for all samples. Since the porosity of the obtained ceramic samples is about 40%, the cells penetrated partially into the pore space of the samples. This study is a pilot in this direction and does not consider the complexity of the interaction of a three-dimensional microenvironment, but concerns the possibility of analyzing metabolic activity using FLIM data. To compare the results of the interaction of cells with ceramic scaffolds obtained using FLIM, the MTT test was used to assess the viability of cells incubated on the ceramic surface.

### 3.3. Cell Viability Analysis

The cytotoxicity of porous ceramics was determined using a standard method—MTT test (colorimetric test) [42]. The MTT test is based on the measurement of cell viability through metabolic activity using a colorimetric test. MTT (3-(4,5-dimethylthiazole-2-yl)-2,5-diphenyltetrazolium bromide) is a yellow water-soluble tetrazolium salt, which is metabolically reduced by mitochondrial succinate dehydrogenase (SDH) from viable fibroblasts, producing formazan products (blue–violet salt), which cannot cross plasma membranes and accumulates in cells. The number of viable fibroblasts correlates with the color intensity determined photometrically by dissolving formazan in dimethyl sulfoxide (DMSO). To assess cell proliferation on the ceramic surface, fibroblast viability analysis was performed after 24 and 48 h of incubation. The optical density of dissolved formazan was measured using a Multiscan FC photometer (ThermoFisher Scientific, Dreieich, Germany) at a wavelength of 570 nm. As a control, a glass coverslip with high adhesive surface properties, which favor active cell proliferation, was used.

### 3.4. Fixation of Fibroblast Culture on Ceramic

The fixation of fibroblasts on the samples was carried out by osmosis. For this purpose, fibroblasts were fixed with 2.5% glutaraldehyde (Serva, Heidelberg, Germany) in a 0.1 M cacodylate buffer (Serva, Heidelberg, Germany) for 30 min. After the fixation was completed, the fixator was washed with 0.2 M cacodylate buffer for 20 min and osmated with 1% OsO_4_ solution (SPI-CHEM, West Chester, PA, USA) in 0.1 M cacodylate buffer for 30 min in the dark, followed by washing with 0.2 M cacodylate buffer for 20 min [43,44,45]. Then, the samples were dehydrated in a series of graduated ethanol solutions (50, 70, 80, 90, and 96 vol.%), followed by acetone and hexane.

### 3.5. Fibroblast Morphology

To visualize cells on glass (control measurements), an inverted Zeiss AxioVert A1 microscope (Zeiss, Oberkochen, Germany) was used.

A scanning electron microscope (SEM) LEO EVO 50 (Zeiss, Oberkochen, Germany) was used to describe the morphology of cells cultured on the surface of the studied ceramics. The samples were then dried and placed in a vacuum post for copper deposition to prevent charging during SEM imaging. SEM is a research method based on the use of an electron microscopy to obtain high-resolution images of objects. It is used to analyze cell cultures, as it allows for a more detailed view of cell surface morphology than a conventional optical microscope. SEM can also be used to study the surface of materials, including medical implants, to assess their compatibility with tissues and cells. In this study, SEM was used to analyze the morphological changes in cells during their proliferation on glass and ceramic surfaces. SEM images of the surface of ceramic samples with cultured cells will provide additional information about the morphology and the interaction of cells with ceramic materials.

### 3.6. FLIM Data Examination and Analysis

For each sample, 5 images were obtained from different points. Two-photon-excited fluorescence intensity and FLIM images were recorded using two-photon microscope (MPTflex, Jena, Germany). Fluorescence of fibroblast fluorophores was carried out under laser excitation at wavelengths of 760 nm and 830 nm. Two-photon excitation of NAD(P)H occurred at a wavelength of 760 nm, and FAD at a wavelength of 830 nm (the number of measurements is shown in Table 1). To ensure that the autofluorescence intensity is not less than 200 photons per pixel, the power and acquisition time were offset to 7 mW and 12 s, respectively. FLIM images were recorded on a 128 × 128 pixel^2^ matrix, and the image size was 100 × 100 μm^2^. The fluorescence lifetime of fluorophores was carried out using software package “Becker&Hickl” [46,47]. FLIM data processing was carried out in the SPCImage 8.6 NG software.

**Figure 7:**
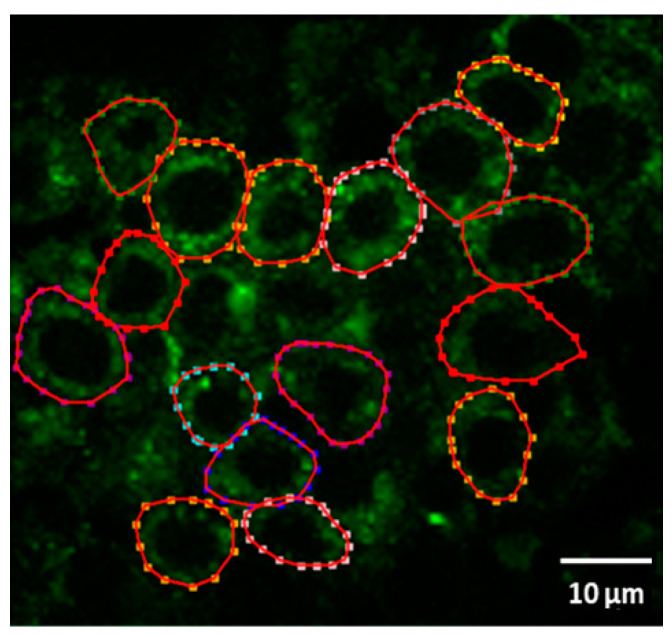
Isolation of fibroblasts using the Python program-based developed script.

Fibroblasts were manually isolated on the FLIM image using a Python program-based developed script that allowed segment (cell) selection in the image, counting their number, and superimposing them on the recorded data (the number of cells is shown in Table 1). Since the selection was carried out manually, the selection error was minimized, and the cells were selected only in the center where they are clearly visible and have sufficient intensity. Example of fibroblast boundary selection on image is shown in Figure 7.

It is known that fluorescence lifetimes and amplitudes of NAD(P)H and FAD are sensitive to metabolism changes in cells [48]. The most common optical method for metabolic imaging is the “redox ratio” [21]. The redox ratio was defined as the fluorescence intensity of FAD divided by the fluorescence intensity of NAD(P)H, that is, without using information about the lifetime. Another way to assess the metabolic status is to analyze the FLIM decay curves. Two-exponential approximation allows us to estimate FLIM decay curves, and phasor plot helps us to visualize contribution of different fluorophores [49,50]. Two-exponential approximation is used to fit the fluorescence lifetime decay, which is calculated by the formula:

(1) $$I\left(t\right)=a_{1}e^{-t/\tau_{1}}+a_{2}e^{-t/\tau_{2}},$$

where $\tau_{1}$ is the short fluorescence lifetime, $\tau_{2}$ is the long fluorescence lifetime, and $a_{1}$ and $a_{2}$ are the corresponding amplitudes of the fitting components [51].

The mean fluorescence lifetime ($\tau_{m}$) was calculated as follows:

(2) $$\tau_{m}=(a_{1}\tau_{1}+a_{2}\tau_{2})/(a_{1}+a_{2})$$

This parameter is used to characterize FLIM data. In the time-domain FLIM, the number of photons is measured after the excitation pulse, and the $\tau_{m}$ is calculated by graph slope of logarithm $I\left(t\right)$, which is called the decay curve [52]. Ratio $a_{1}$/$a_{2}$ is calculated for each pixel in the image.

The phasor plot can also be employed for analyzing time-domain data [53]. The phasor plot is a graphical representation of all the raw FLIM data in a vector space. The projection of the FLIM data into the phasor plot is carried out by the Fourier transform:

(3) $$T\left(\omega \right)=\int_{-\infty }^{\infty }I\left(t\right)\cos\left(\omega t\right)dt-j\int_{-\infty }^{\infty }I(t)\sin(\omega t)dt,$$

where j is imaginary unit, ω is frequency, and $I\left(t\right)$ is a FLIM curve. In this case, the imaginary and real parts of the first Fourier component of the fluorescence decay curves of each pixel are mapped as points in the phasor plot. In this approach, we consider the nonnegative parameter *t*, normalized by the total number of photons equal to $\int_{0}^{\infty }I(t)$, and the axis of abscissa and ordinate represent symbols g and s, respectively, where:

(4) $$g\left(\omega \right)=\frac{\int_{0}^{\infty }I\left(t\right)\cos\left(\omega t\right)dt}{\int_{0}^{\infty }I(t)},\;s(\omega )\frac{\int_{0}^{\infty }I(t)\sin(\omega t)dt}{\int_{0}^{\infty }I(t)}.$$

The real part (g) and an imaginary part (s) are the co-ordinates of the phasor transform at two harmonics of the laser repetition frequency, respectively, for each pixel of the image. The location of the pixel in the phasor plot depends on the amplitude and lifetime of the decay function in the phasor space.

### 3.7. Statistical Analysis

The Henze–Zirkler multivariate test was used to assess the normality of the distribution [54]. The Shapiro–Wilk test [55] was used to determine significant differences between the samples. The Mann–Whitney test [56] was used to determine significant differences between samples and to assess the significance of the difference in the optical density of cells in experimental wells compared to controls. The results of the cell viability assays were presented as mean ± standard deviation (STD); significant difference was *p* < 0.05.

## 4. Discussion

The phasor plot representation of the two-photon-excited FLIM data allows the real-time analysis of the metabolic status of living cells [57]. Differences in the distribution of the NAD(P)H and FAD concentrations in both free and protein-bound forms in 3T3 fibroblasts were revealed.

**Figure 8:**
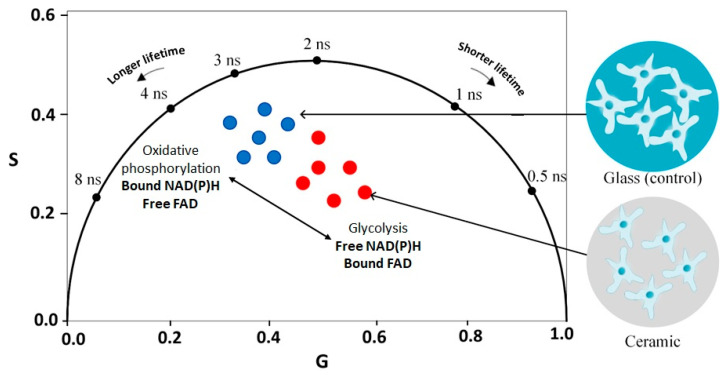
Phasor plot representation of FLIM data of fibroblasts incubated on a control glass (blue) and ATZ and Y-TZP ceramics (red): the shift towards shorter lifetimes (free NAD(P)H and protein-bound FAD forms) corresponds to glycolysis, and the shift towards longer lifetimes (protein-bound NAD(P)H and free FAD forms) corresponds to oxidative phosphorylation.

On the phasor plot presented for NAD(P)H, the fluorescence lifetime has a shift towards the free form of NAD(P)H (short lifetimes), which is observed in fibroblasts when glycolysis predominates over oxidative phosphorylation (long lifetimes, protein-bound form of NAD(P)H) [23,27], as schematically presented in Figure 8. It is confirmed by the results presented in Table 1—all compared groups are characterized by the predominance of the short decay component a_1_, which indicates the presence of the free form of NAD(P)H.

The distribution of bound/free forms of FAD was also analyzed for all groups. According to the phasor plot in Figure 2c, it can be concluded that, in all groups, there is a predominance of the protein-bound form of FAD, which is indicated by a shift towards a shorter fluorescence lifetime. The phasor plot of the protein-bound FAD is present in all compared groups, which is consistent with the results in Table 2.

Based on the distribution data on the phasor plot, fibroblasts in which the bound form of FAD presumably predominates are also observed in the control group. In the ATZ and Y-TZP groups, a change in the FAD/NAD(P)H ratio is observed, but it is not statistically significant compared to the control (see Figure 3). The noted trend towards a slight shift in the ATZ and Y-TZP groups may indicate a shift in the metabolic status of fibroblasts towards increased glycolysis, i.e., the increased metabolic activity of fibroblasts incubated on ATZ and Y-TZP ceramics compared to the control glass. The amplitude ratio $a_{1}$/$a_{2}$ of the decay components was analyzed (Figure 1a and Figure 2a), which indicates the distribution of concentrations of free/bound NAD(P)H and bound/free FAD. An observed shift towards a higher $a_{1}$/$a_{2}$ for NAD(P)H (Figure 1a) indicates a shift in metabolism towards glycolysis [57], which is confirmed by the percentage ratio of the amplitudes of the decay components, as presented in Table 1. Accordingly, the shift of cell metabolism towards glycolysis may indicate that cells experience some state of hypoxia when incubated on porous ceramic structures. This may be due to the growth of cells inside the pores of the ceramic structure. Under hypoxic conditions, glycolysis increases to compensate for the decrease in the intensity of mitochondrial respiration [27].

The average fluorescence lifetimes ($\tau_{m}$) of the NAD(P)H and FAD cofactors were also measured, which is shown in Figure 1b and Figure 2b. However, as can be seen from Table 1, the $\tau_{m}$ for Y-TZP is slightly higher compared to the control and ATZ. As can be seen from Table 2, the $\tau_{m}$ parameter is higher in ATZ compared to the control and Y-TZP.

The statistically significant difference between the control group and ATZ obtained in the MTT test indicates that the zirconia ceramic has weak surface adhesive properties, while the low cell viability might not be due to the material toxicity, but to the inability of the cells to adhere to the substrate (see Figure 4). Based on the results of the MTT test, it can be assumed that all the investigated ceramic structures are non-cytotoxic and suitable as bioinert materials for osteoimplants. To assess the morphology of the cells, the SEM method was used in combination with light microscopy. The 3T3 fibroblasts incubated on control glass and ceramic samples ATZ and Y-TZP have a flattened fusiform shape with elongated lamellipodia (Figure 5). This indicates that the material is non-toxic and does not cause changes in the morphology of 3T3 cells. A less pronounced proliferation was noted for the ATZ sample, which we attribute to the adhesive properties of the ATZ material, since all other results indicate normal cell parameters (SEM and FLIM).

## 5. Conclusions

In this paper, we propose an in vitro method for diagnosing the effects of osteoimplant ceramics on the surrounding tissue fibroblasts, based on the analysis of the redox status of fibroblasts by two-photon-excited autofluorescence and FLIM. Verification was carried out using the MTT test and SEM imaging.

Using two-photon microscopy with FLIM, it is possible to estimate the redox coefficient and fluorescence lifetime of NAD(H)P and FAD fluorophores. The use of FLIM and the phasor approach made it possible to evaluate the ratio of free and bound forms of NAD(P)H and FAD in fibroblasts of the 3T3 line, as well as to identify changes in the parameters of the fluorescence of metabolic cofactors depending on the ceramics on which the cells were incubated. FLIM allows us to analyze the metabolic characteristics of cells without additional preparation and staining.

Using the MTT test in combination with scanning electron microscopy allows us to make a conclusion about the viability of cells, and their proliferation and shape, which is an important criterion for assessing the cytotoxicity of osteoimplants. The low viability of the 3T3 line on ATZ ceramics is associated with poor cell adhesion upon initial seeding. When cells are incubated on samples, growth dynamics are observed, which indicates favorable conditions for proliferation. The methods and approaches to data analysis presented here can be applied in the case when it is necessary to assess the metabolic state of cells that are incubated on material structures used as osteoimplants.

## Figures and Tables

**Table 1:** Optical characteristics of the 3T3 fibroblasts (two-photon excitation at 760 nm; fluorophore is NAD(P)H).

	Optical Characteristics (Mean ± STD)					
	$a_{1}$ (%)	$a_{2}$ (%)	$a_{1}$ $/a_{2}$	$\tau_{1}$ (ps)	$\tau_{2}$ (ps)	$\tau_{m}$ (ps)
Control	74 ± 11	26 ± 11	3.48 ± 2.1	459 ± 93	2595 ± 762	774 ± 161
ATZ	75 ± 4	25 ± 4	3.82 ± 1.6	494 ± 110	2498 ± 409	766 ± 178
Y-TZP	73 ± 9	27 ± 9	3.55 ± 1.8	552 ± 134	2728 ± 517	881 ± 260

**Table 2:** Optical characteristics of the 3T3 fibroblasts (two-photon excitation at 830 nm; fluorophore is FAD).

	Optical Characteristics (Mean ± STD)					
	$a_{1}$ (%)	$a_{2}$ (%)	$a_{1}$ $/a_{2}$	$\tau_{1}$ (ps)	$\tau_{2}$ (ps)	$\tau_{m}$ (ps)
Control	83 ± 6	17 ± 5	4.3 ± 2.1	245 ± 117	1512 ± 491	438 ± 161
ATZ	82 ± 13	18 ± 13	5.1 ± 2.3	299 ± 120	1804 ± 408	594 ± 243
Y-TZP	87 ± 10	13 ± 9	4.6 ± 2.2	214 ± 145	1364 ± 538	434 ± 245

**Table 3:** Number of samples, number of measurements, and cell density for each group of cells.

	Number of Samples	Number of Measurements		Number of Cells		Cell Density on the One FLIM Image (Cells/0.1 cm^2^)
		Excitation at 760 nm	Excitation at 830 nm	Excitation at 760 nm	Excitation at 830 nm	
Control	10	56	56	1130	306	21 ± 12
ATZ	5	17	17	185	81	19 ± 10
Y-TZP	5	9	9	229	59	15 ± 8

## Data Availability

The data presented in this study are available upon request from the corresponding author. The data are not publicly available due to privacy or ethical restrictions.
